# Ulcerative colitis reporting across medications: FAERS analysis and cross-species transcriptomic triangulation

**DOI:** 10.3389/fimmu.2026.1827266

**Published:** 2026-10-09

**Authors:** Huan Long, Caihua Duan, Jiusi Liu, Nanxi Xiao, Jiang Huang, Huilei Zeng, Zhenling Gao, Yao Chen

**Affiliations:** 1 Department of Emergency, Ji’an Central People’s Hospital, Jizhou District, Ji’an, Jiangxi, China; 2 Department of Gastroenterology, Ji’an Central People’s Hospital, Jizhou District, Ji’an, Jiangxi, China; 3 Department of Gastrointestinal Surgery, Ji’an Central People’s Hospital, Jizhou District, Ji’an, Jiangxi, China; 4 Department of Cardiology, Ji’an Central People’s Hospital, Jizhou District, Ji’an, Jiangxi, China

**Keywords:** colonic transcriptomics, drug safety, FAERS, IL-17, pharmacovigilance, ulcerative colitis

## Abstract

**Background:**

Ulcerative colitis (UC) is reported with medications from different therapeutic classes. We examined these reporting associations together with recorded clinical context and tested whether experimental cytokine-blockade signatures align with human UC mucosal expression.

**Methods:**

FAERS reports from 2004Q1–2024Q4 were restricted to health professionals and primary-suspect medications. Ten previously identified candidates were evaluated using unique report–generic counts, reporting odds ratios (RORs), proportional reporting ratios, and a supportive Bayesian information component. Sensitivity analysis excluded complete reports recording UC as an indication. Directional signatures from five antibody-versus-isotype contrasts in a 42-sample mouse colitis study were scored in two external human cohorts (87 UC/21 controls and 74 active UC/11 controls), using 12,109 common one-to-one orthologs. Ten primary score comparisons underwent joint Benjamini–Hochberg correction.

**Results:**

Among 7,300,798 eligible FAERS reports, 11,352 included UC. Eight candidates met the specified reporting criteria; seven retained support after UC-indication exclusion. Secukinumab and isotretinoin had 204 and 130 UC reports, with RORs of 4.49 (95% CI 3.90–5.16) and 3.79 (3.19–4.51). Valganciclovir decreased from nine to three reports after exclusion and became imprecise. In human mucosa, experiment-2 IL-17A and IL-17RA blockade signatures scored lower in UC in both cohorts; experiment-1 IL-17RA showed a positive difference in only one cohort. IL-23p19 and IL-12/23p40 blockade signatures scored lower in UC in both cohorts. These patterns persisted across the planned signature-size sensitivity analysis.

**Conclusion:**

Event-centered analysis differentiated reporting associations by their indication context. External experimental and patient data did not support a uniform UC-like transcriptional pattern following IL-17 blockade, refining the mechanistic interpretation of the reporting signals. The findings identify priorities for drug-specific clinical investigation without estimating incidence or establishing causality.

## Introduction

Ulcerative colitis (UC) is a chronic inflammatory disorder of the colon, diagnosed through clinical assessment supported by endoscopic and histological findings. Medication-associated intestinal injury can resemble or complicate inflammatory bowel disease (IBD), but the spectrum of drug-related colitis is broader than UC and includes distinct pathological processes. This distinction is important when interpreting adverse-event terminology without clinical records (1, 2).

Spontaneous adverse-event reports provide a means of examining medication and event co-reporting across therapeutic settings. An event-centered approach can bring together reports that would otherwise be considered in separate drug-specific analysis. Its interpretation depends on consistent case counting, medication-name handling, and the choice of comparison reports. A medication used to treat UC or its complications may be reported alongside UC because of the underlying disease. Reporting frequency also reflects missing information, publicity, and reporting practices. FAERS has no denominator of all treated patients and cannot independently establish whether a medication caused an event (3).

We investigated UC reporting across therapeutic settings and examined ten candidates identified in the initial pharmacovigilance screen. Recorded indications and treatment timing were used to distinguish clinically different reporting contexts. We then focused on IL-17 inhibition, represented by the most frequently reported candidate, secukinumab, and the related IL-17A/F inhibitor bimekizumab. By combining published antibody-intervention experiments with two external human UC cohorts, we tested whether the direction of the experimental colonic expression changes translated consistently to human disease. IL-23p19 and IL-12/23p40 blockade provided mechanistic comparators. This design addresses the relationship between a reporting signal and a disease-relevant molecular pattern, rather than assuming that a known drug target establishes that relationship (4–6).

## Methods

### Data sources and report population

FAERS reports were retrieved on 13 September 2026 through easyFAERS version 0.12.0 using its default cleaned database route, druginfo. Reporting quarters were restricted to 2004Q1–2024Q4 and reporter occupation to physician (MD), pharmacist (PH), or other health professional (OT). The reporting-quarter restriction describes the database reporting period, rather than a clinically adjudicated date of UC onset. The comparison population used the same period and reporter filters. All statistical analysis were performed in R 4.4.2.

We used the provider-deduplicated demographic table. Within the UC population, each primaryid report identifier and each CASEID case identifier occurred once. Related tables were linked by primaryid, and all 84 reporting quarters were represented. The provider’s complete case-version history was unavailable, and distinct case identifiers could not be confirmed to represent distinct patients. The database snapshot identifier and MedDRA release were not returned by the interface.

The event definition was the exact reaction preferred term COLITIS ULCERATIVE in the reaction table. Broader colitis terms and treatment indications were not used to define UC events. A report carrying this term was considered a UC report, without assuming newly diagnosed UC, confirmed histology, or medication causation. The source tables comprised demographic, drug, reaction, indication, therapy, outcome, and report-source information. The cohort and sensitivity-analysis flow are shown in **Figure 1**, with further counting details in **Supplementary Table S1**.

**Figure 1:**
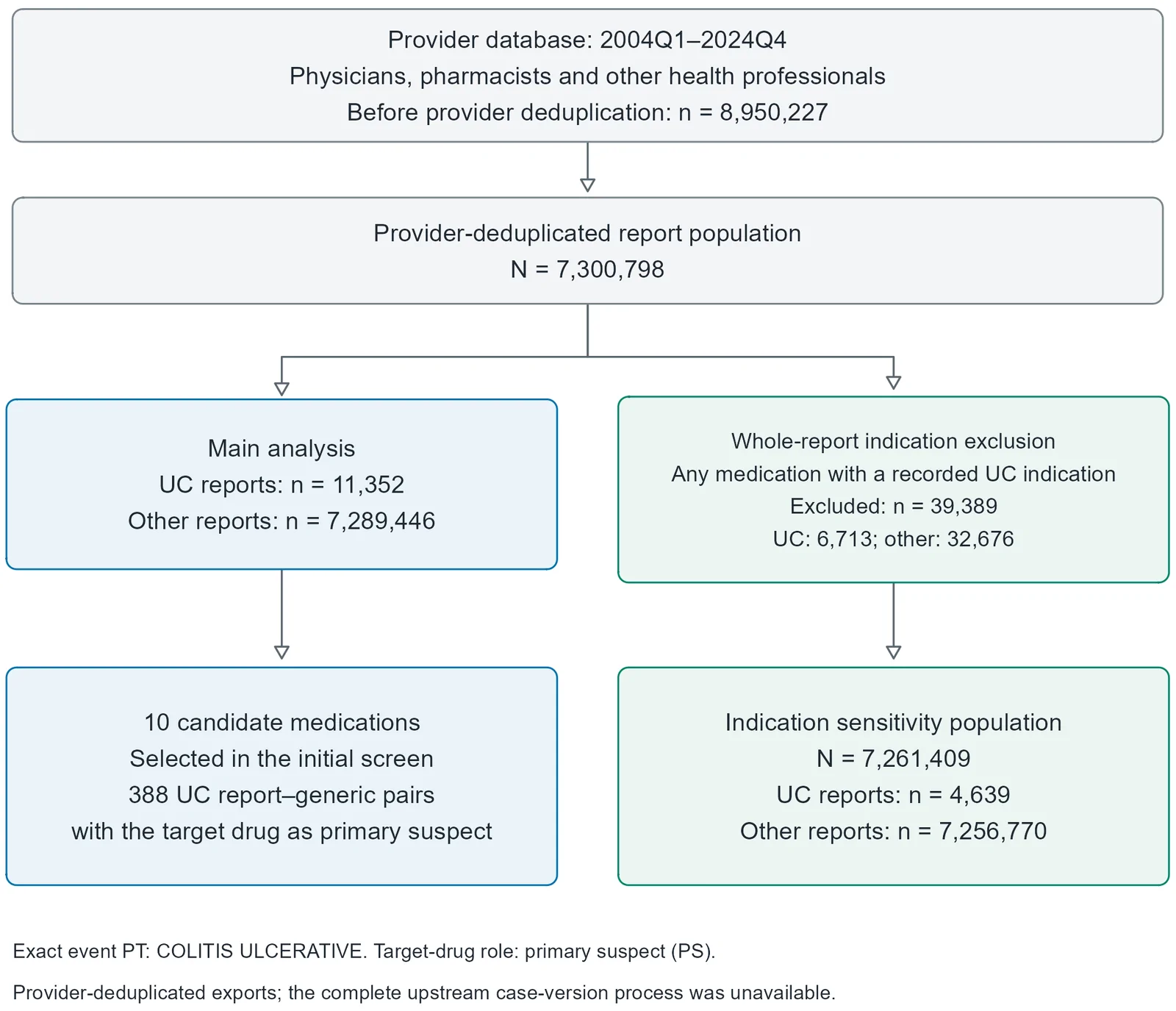
Cohort and indication-sensitivity flow. All counts use the same reporting-quarter and reporter filters. The provider performed source deduplication; the complete case-version process could not be independently reconstructed. The indication exclusion applies to complete reports in the entire background, including UC and non-UC reports.

### Medication definitions and counting units

The ten candidate medications were bimekizumab, calcium carbonate, efalizumab, encorafenib, erdosteine, hydrochlorothiazide, isotretinoin, pentosan polysulfate, secukinumab, and valganciclovir. Candidate-specific analysis were restricted to these medications, which were identified in the initial pharmacovigilance screen. All ten were retained, including sparse or non-estimable results. The frozen query vocabulary contains exact generic, ingredient/salt, and supported brand terms, including the regulator-confirmed RAPTIVA alias for efalizumab. Unmatched names and separately named combination products were not added through fuzzy matching. A recorded name match identifies the reported medication but does not isolate its effect from other ingredients or co-medications. The exact terms and mapping evidence are supplied in **Supplementary Table S2** and the source workbook; coverage of all possible international brand names is not claimed.

Medication exposure required a primary-suspect role (PS) and an exact match of the recorded drug name or active-ingredient field to the frozen query vocabulary. For each medication, unique primaryid values were counted once, even if the report contained multiple matching brand names or medication sequences. The same vocabulary and PS rule were applied to UC reports and the full comparison population. Other medication roles remained available for contextual descriptions. A report could contribute to several medication-specific analysis, but never more than once to a given report–generic pair. Reports, case identifiers, medication sequences, and report–event pairs are consequently distinct units.

### Statistical analysis

For each medication, a denoted exposed UC reports, b exposed reports without the UC reaction term, c UC reports without that medication in a PS role, and d all remaining eligible reports (**Table 1**). Background and exposure totals were obtained with the same filters through EFun, and UC counts were calculated from unique report identifiers. ROR was calculated as ad/(bc), and PRR as [a/(a+b)]/[c/(c+d)]. Their 95% confidence intervals were exponentiated log-scale intervals. The ROR criterion was a ≥ 3 and a lower 95% confidence limit greater than 1. The PRR criterion was a ≥ 3, PRR ≥ 2, and Pearson chi-square ≥ 4 (**Table 2**) (7).

**Table 1:** Report-level contingency definitions.

PS exposure to candidate	UC reaction present	UC reaction absent
Yes	a	b
No	c	d

Each report is counted once per candidate medication. UC denotes the exact reaction preferred term COLITIS ULCERATIVE. The non-exposed category means no matched candidate medication in a primary-suspect (PS) role. It does not establish the absence of all use of that medication. N = a + b + c + d.

**Table 2:** Estimators and exploratory signal conventions.

Quantity	Calculation and interpretation	Flag
ROR	ad/(bc); 95% CI = exp[ln(ROR) ± 1.96 √(1/a + 1/b + 1/c + 1/d)]	a ≥ 3 and lower 95% limit > 1
PRR	[a/(a+b)]/[c/(c+d)]; 95% CI = exp[ln(PRR) ± 1.96 √(1/a − 1/(a+b) + 1/c − 1/(c+d))]	a ≥ 3, PRR ≥ 2, Pearson χ² ≥ 4
Posterior IC and IC025	Specified Bayesian posterior mean; IC025 = mean − 1.959964 × posterior SD. Normal approximation; equations in **Supplementary Methods**.	Supportive only: a ≥ 3 and IC025 > 0
Fisher exact interval	Conditional odds-ratio estimate and two-sided 95% interval, supplied for sparse-data comparison.	Supplementary precision check

ROR, reporting odds ratio; PRR, proportional reporting ratio; IC, information component. Agreement among these calculations is not independent validation. Empty margins are reported as non-estimable; no automatic 0.5 correction was used.

As a supportive analysis, we computed a Bayesian information-component posterior mean using the specified Bate model and Gould moment formulation. IC025 was the posterior mean minus 1.959964 posterior standard deviations, a normal approximation to the lower 2.5% bound. This quantity is distinguished throughout from the posterior mean; it is not an exact posterior quantile. The supportive criterion was a ≥ 3 and IC025 > 0. The explicit priors, moment equations, and executable R function are supplied in the **Supplementary Methods**. Implementation was cross-checked against PhViD 1.0.8 with its non-Monte-Carlo BCPNN calculation. Sparse-count normal approximations remain a limitation (8, 9).

Signal estimates were interpreted as exploratory reporting associations. The candidates were selected in the initial screen, so their signal flags do not represent multiplicity-controlled discoveries from an unrestricted all-drug analysis. Agreement between algorithms using the same reports is not independent validation. Fisher exact conditional odds-ratio intervals were supplied as a sparse-data check. Log-Wald estimates were omitted when a required cell was zero, and all effect estimates were omitted for an empty exposure margin; no automatic continuity correction was applied.

Demographic summaries used one row per UC report. Missing counts and percentages used all 11,352 UC reports as the denominator; percentages restricted to available observations are separately identified. Age and weight were summarized using the provider’s numeric exports, interpreted as years and kilograms because the software’s category definitions and exported value patterns support prior normalization. The retained original unit codes were not applied a second time. As the upstream conversion program was unavailable, sensitivity summaries restricted to YR-coded age and KG-coded weight are also provided. Broad plausibility checks used age 0–120 years and weight greater than 0 to 500 kg. No nonmissing value was excluded by these limits.

The outcome export contained one retained category per report. We describe that category and its missingness without treating it as a reconstruction of all coexisting outcomes or attributing it to UC or a particular drug. The analysis did not estimate multivariable-adjusted medication effects.

### Indication sensitivity and clinical context

An explicit UC indication was defined as COLITIS ULCERATIVE in any indication record belonging to a report, regardless of which medication that indication described. A sensitivity analysis excluded those entire reports from both the UC-event and comparison populations. For each medication, counts in the UC-indication subset were obtained using the same exposure definition and subtracted from the corresponding cells of the main contingency table. This excluded complete reports, including those with both UC and other indications.

Reports with missing or unknown indications remained in the analysis and were not interpreted as confirmed absence of pre-existing UC. Both PRODUCT USED FOR UNKNOWN INDICATION and DRUG USE FOR UNKNOWN INDICATION were classified as unknown when describing completeness. In addition to report-level indication overlap, indications were linked to the target medication by primaryid and DRUG_SEQ. Concomitant and other reported medications were tabulated to describe clinical context, particularly for valganciclovir. These descriptions and the indication exclusion do not constitute comprehensive adjustment for concomitant treatments, disease severity, or unrecorded indications.

### Recorded time to onset

Target PS drug records were linked to therapy records using both primaryid and DRUG_SEQ, then to the report-level event and FDA receipt dates. The recorded interval was EVENT_DT minus START_DT. We accepted only complete, valid calendar dates and did not impute partial dates. Missing or invalid dates, absent linked therapy records, negative intervals, dates after FDA receipt, and ambiguous multiple treatment-start dates were excluded. Zero-day intervals were retained. A report–generic pair required valid linked records and one unambiguous start date. Exclusion reasons and usable-date denominators are supplied in **Supplementary Table S4**. Median and interquartile range (IQR) were reported when at least five report–generic pairs had evaluable dates. The report-level event date was not independently adjudicated as the onset date of UC.

### External experimental and human transcriptomic analysis

To test molecular translation beyond FAERS, we reanalyzed GSE72212, a published colitis experiment in Abcb1a-deficient mice challenged with Helicobacter bilis, and two independently sourced human colonic datasets. GSE72212 contains 42 samples from two experiments. Each antibody was compared with the isotype group within its own experiment: anti-IL-17A (5 versus 4) and anti-IL-17RA (5 versus 4) in experiment 2, and anti-IL-17RA (5 versus 3), anti-IL-23p19 (5 versus 3), and anti-IL-12/23p40 (3 versus 3) in experiment 1. All groups within each experiment contributed to variance estimation. These were experimental cytokine-blocking antibodies, not administrations of the clinical candidate medicines (4).

The human comparisons were GSE87466 (87 UC and 21 healthy control colonic biopsies) and GSE75214 (74 active UC and 11 normal colon biopsies). The 23 inactive UC colon samples in GSE75214 were reserved for descriptive activity context; CD and ileal samples were excluded from the main comparison. GSE59071 was excluded because its colon cohort overlaps GSE75214. Each dataset was analyzed separately without pooling or between-study batch correction (5, 6).

Official deposited matrices and platform annotations were matched by GSM identifiers. Human RMA log2 values were used as deposited. Positive mouse MAS5 intensities were log2 transformed and quantile normalized separately within each experiment. Ambiguous multi-gene and nonstandard locus assignments were excluded. Multiple probes for a gene were collapsed by the highest mean across all deposited samples, independently of group effects. Official MGI homology groups were restricted to globally unambiguous one-to-one human–mouse pairs. The common measured universe was fixed before signature selection. Mouse contrasts and separate human UC-versus-control contrasts used ordinary empirical-Bayes limma 3.62.2 models (10, 11).

For each mouse contrast, the 250 highest positive and 250 lowest negative moderated t statistics within the common universe defined a directional signature. In each human sample, expression was converted to percentile ranks across that universe; the score was the mean rank of mouse-up genes minus the mean rank of mouse-down genes. Higher scores indicate greater alignment with the experimental direction. Within-cohort UC-minus-control differences used linear models with HC3 robust standard errors and 95% t intervals. All ten primary comparisons underwent joint Benjamini–Hochberg correction. Signature sizes of 100 and 500 genes per direction generated 20 planned sensitivity comparisons, corrected as a separate family. These analyses were exploratory and were not preregistered. The selection rules were fixed before the expression results were calculated.

All eight preselected epithelial/inflammatory context genes (PIGR, MUC2, TJP1, OCLN, CLDN1, CXCL1, CXCL2 and LCN2) were retained in the supplementary results, with unavailable orthologs marked explicitly. Official prescribing information and ChEMBL mechanism records were also examined for every FAERS candidate, distinguishing direct human targets, metabolite-mediated action, viral targets and chemical or physiological effects. Full sample flow, preprocessing, scoring and evidence sources are provided in **Supplementary Tables S7**–**S10**.

## Results

### Report population and missingness

The filtered provider population contained 8,950,227 demographic records before provider deduplication and 7,300,798 afterward. The UC extraction included 11,352 unique report identifiers and the same number of unique case identifiers, compared with 7,289,446 reports without the target reaction term. There were 80,889 drug rows and 80,369 unique report–drug-sequence pairs in the UC export. One UC report had no PS drug. These quantities are not interchangeable counts of patients or drug-caused UC events (**Figure 1**; **Supplementary Table S1**).

Age was available for 7,892 reports, with a median of 43 years (IQR 26–60); weight was available for 3,466, with a median of 67 kg (54–81). Age, weight, and sex were missing in 3,460 (30.48%), 7,886 (69.47%), and 1,197 (10.54%) reports, respectively. Female and male sex were recorded in 5,357 and 4,798 reports, representing 47.19% and 42.27% of all reports, or 52.75% and 47.25% of those with recorded sex (**Table 3**). Restriction to retained YR and KG unit codes gave medians of 43 years (26–59) and 66.6 kg (53.6–81), respectively.

**Table 3:** UC report characteristics and explicit denominators.

Characteristic	Available or category n	Missing n	Summary or percent
All UC reports	11,352	—	100%
Age	7,892	3,460 (30.48%)	43 (26–60) years
Weight	3,466	7,886 (69.47%)	67 (54–81) kg
Recorded sex	10,155	1,197 (10.54%)	—
Female	5,357	—	47.19% total; 52.75% known
Male	4,798	—	42.27% total; 47.25% known
Physician reporter	8,671	0	76.38%
Pharmacist reporter	669	0	5.89%
Other health professional	2,012	0	17.72%
Retained outcome code	9,669	1,683 (14.83%)	One retained category/report
Hospitalization category	4,074	—	35.89% of all reports
Death category	268	—	2.36% of all reports

Continuous summaries are median (IQR). Total percentages use N = 11,352; known-sex percentages use n = 10,155. Age and weight use provider numeric exports interpreted as years and kilograms; the original unit-code sensitivity summaries are in **Supplementary Table S1**. The retained outcome code does not reproduce every original coexisting outcome and is not attributed to UC or a medication. Minor percentage totals may differ from 100 because of rounding.

Physicians submitted 8,671 reports, pharmacists 669, and other health professionals 2,012. Annual counts increased from 73 in 2004 to 1,386 in 2024, with year-to-year variation (**Figure 2**). The leading reporter countries were the United States (3,070) and Canada (2,737); these are reporter locations, not necessarily patient residences. Among provider-retained outcome categories, 5,097 reports carried other medically important events, 4,074 hospitalization, and 268 death; 1,683 had a missing retained category. These outcome codes do not establish attribution to UC or a target medication.

**Figure 2:**
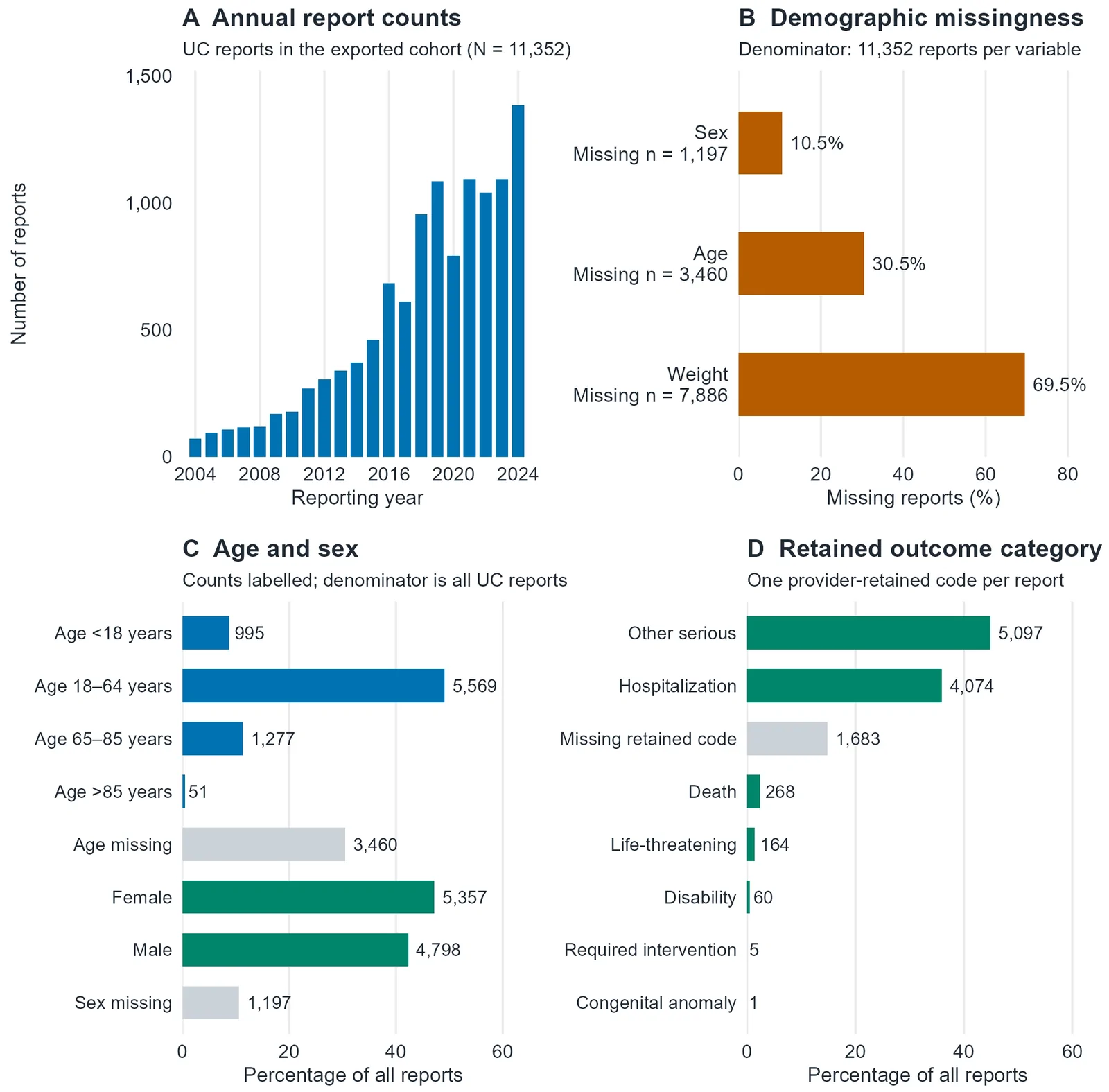
Characteristics of UC reports. **(A)** Annual report counts. **(B)** Demographic missingness. **(C)** Age and sex distribution. **(D)** Retained outcome category. Annual counts and demographic distributions describe the reporting population. Missingness denominators are shown explicitly. The outcome panel describes the single category retained by the provider per report; it is not a complete reconstruction of all original outcomes or an attribution to UC.

### Medication-level reporting associations

Across the ten candidates, the dataset contained 388 PS report–generic pairs. Eight candidates met the specified ROR, PRR, and supportive IC025 criteria: bimekizumab, calcium carbonate, efalizumab, encorafenib, isotretinoin, pentosan polysulfate, secukinumab, and valganciclovir. Hydrochlorothiazide had one qualifying UC report and did not meet the minimum-count criterion. No eligible erdosteine-exposed PS report was identified using the frozen vocabulary, so its estimate was not calculable. **Table 4** presents results for all ten candidate medications.

**Table 4:** Reporting associations for the ten candidate medications.

Generic medication	UC reports a	ROR (95% CI)	PRR (95% CI)	IC mean (IC025)
Bimekizumab	5	7.58 (3.14–18.30)	7.50 (3.14–17.93)	1.72 (0.51)
Calcium carbonate	7	18.51 (8.73–39.25)	18.02 (8.68–37.41)	2.44 (1.39)
Efalizumab	9	3.64 (1.89–7.02)	3.63 (1.89–6.96)	1.45 (0.53)
Encorafenib	12	4.08 (2.31–7.20)	4.06 (2.31–7.14)	1.66 (0.86)
Erdosteine	0	NE	NE	NE
Hydrochlorothiazide	1	0.15 (0.02–1.05)	0.15 (0.02–1.05)	-2.34 (-4.61)
Isotretinoin	130	3.79 (3.19–4.51)	3.77 (3.17–4.48)	1.87 (1.62)
Pentosan polysulfate	11	17.50 (9.61–31.86)	17.06 (9.52–30.58)	2.81 (1.96)
Secukinumab	204	4.49 (3.90–5.16)	4.46 (3.89–5.12)	2.11 (1.91)
Valganciclovir	9	2.68 (1.39–5.15)	2.67 (1.39–5.12)	1.12 (0.20)

All rows use N = 7,300,798 background reports and 11,352 UC reports; exposure totals and a/b/c/d are supplied in **Supplementary Table S3**. Eight rows meet all stated exploratory criteria; hydrochlorothiazide has only one target report, and erdosteine has an empty eligible exposure margin (NE, not estimable). IC025 is a normal approximation, not the IC point estimate. These reporting estimates do not rank absolute clinical risk across medications.

Secukinumab and isotretinoin had 204 and 130 UC reports, with RORs of 4.49 (95% CI 3.90–5.16) and 3.79 (3.19–4.51), respectively. The other positive candidates had between five and twelve reports, with correspondingly limited precision. Calcium carbonate and pentosan polysulfate had seven and eleven reports despite relatively large point estimates. Full counts, RORs, PRRs, posterior IC means, and IC025 values are reported in **Table 4** and **Supplementary Table S3**; **Figure 3** presents ROR intervals. Point-estimate ordering is not a ranking of absolute clinical risk.

**Figure 3:**
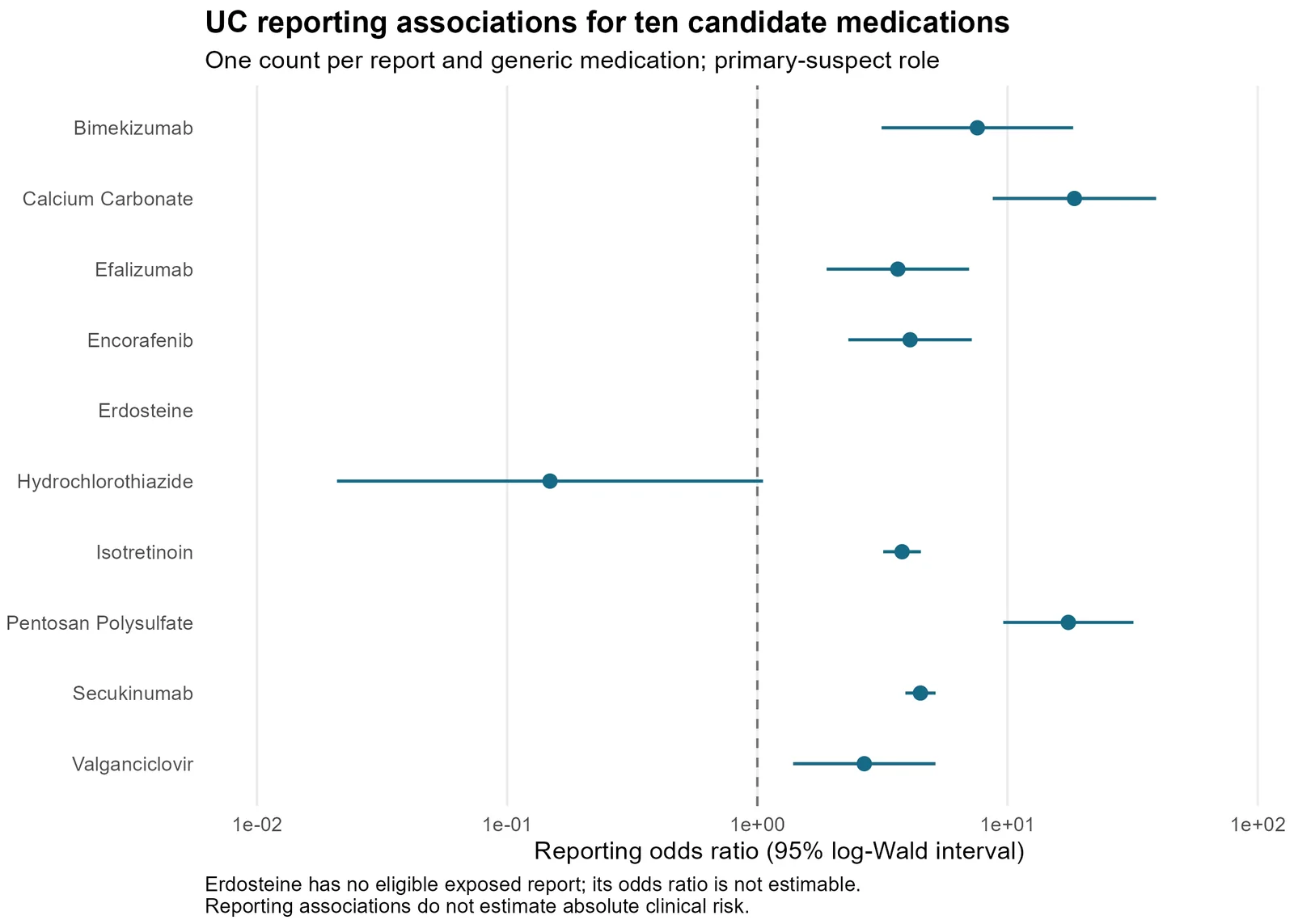
Main reporting odds ratios. Points denote RORs and horizontal lines their 95% log-Wald confidence intervals. All ten candidate medications are displayed; erdosteine has no estimable ROR. The reference line is 1. Full report counts and estimates are in **Table 4**.

### Indication sensitivity and timing

A recorded UC indication occurred in 39,389 background reports, including 6,713 of the UC reports. Excluding those reports left 7,261,409 background reports and 4,639 UC reports. Seven of the eight initially supported candidates retained the three specified flags; valganciclovir did not. Secukinumab retained 201 UC reports with an ROR of 11.06 (95% CI 9.60–12.75), and isotretinoin retained 130 with an ROR of 9.39 (7.88–11.18). Valganciclovir decreased from nine to three reports and had an ROR of 2.18 (0.70–6.78); its Fisher interval also included 1 (**Figure 4**; **Supplementary Table S3**). These increases in some reporting ratios reflect a different comparison distribution and do not imply increased clinical risk after exclusion.

**Figure 4:**
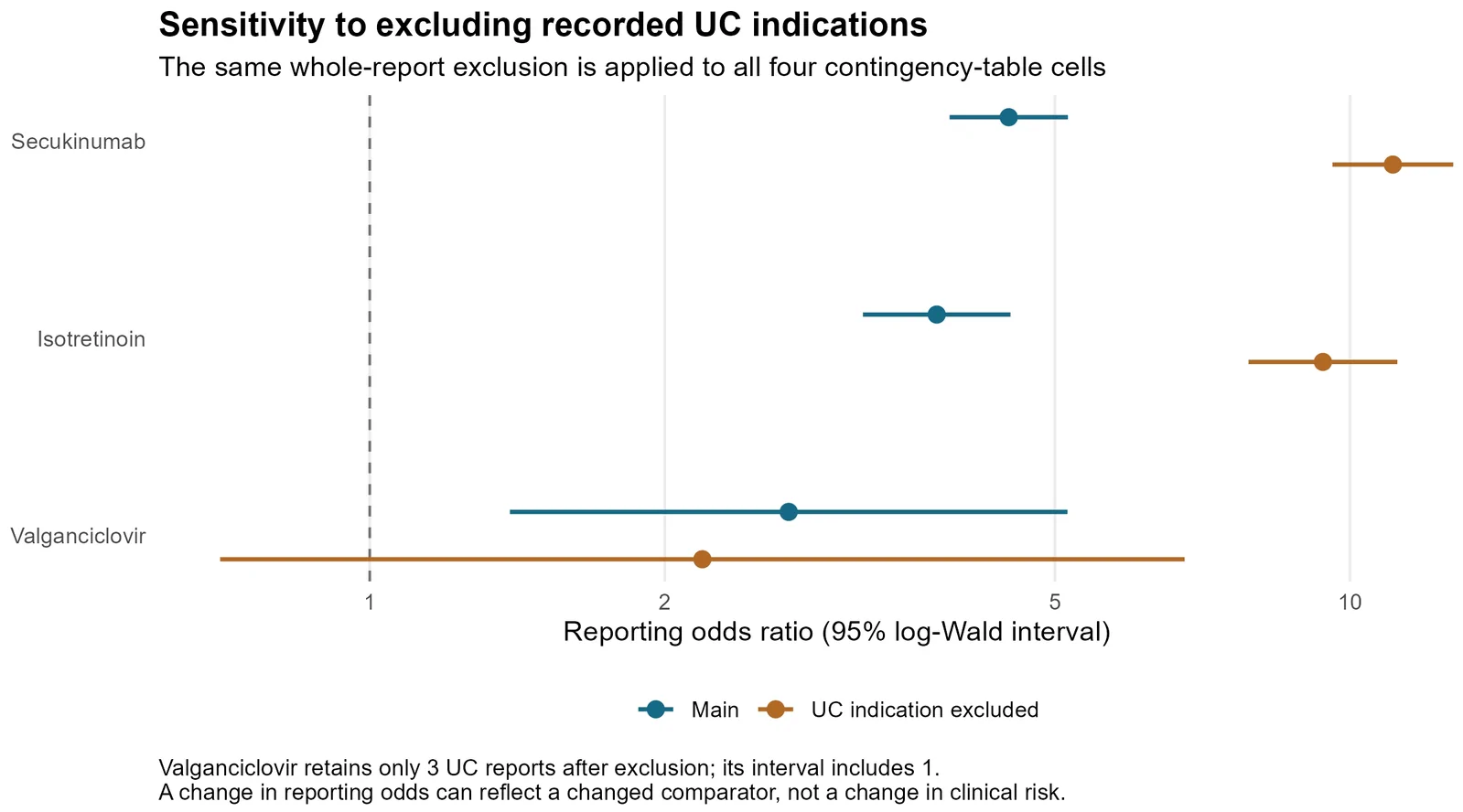
Recorded-indication sensitivity for selected associations. Main and whole-report UC-indication-excluded RORs are shown for secukinumab, isotretinoin, and valganciclovir. Horizontal lines are 95% log-Wald intervals. Full sensitivity results for all ten candidates are in **Supplementary Table S3**. The estimates describe reporting associations without multivariable adjustment.

Six of the nine valganciclovir UC reports recorded UC as an indication for another medication; none linked UC to valganciclovir itself. Valganciclovir-linked indications included cytomegalovirus-related terms in three reports, prophylaxis terms in two, and unknown indication in four. The co-medication profiles included IBD therapies and immunosuppressive treatments. Across all UC reports, 994 had no non-unknown indication recorded, so absence of a recorded UC indication did not establish absence of underlying disease (**Supplementary Table S5**).

Usable linked dates were present for 72 of 204 secukinumab reports (35.29%) and 65 of 130 isotretinoin reports (50.00%). Their median recorded intervals were 92 days (IQR 46–289.25) and 175 days (31–593). Encorafenib had five evaluable reports among twelve (41.67%), with a median of 90 days (58–259), a sparse descriptive result. The remaining candidates had fewer than five evaluable reports and were retained with their available and excluded counts rather than fitted timing distributions (**Supplementary Table S4**; **Supplementary Figure S1**).

### Directional comparison with human UC mucosa

Preprocessing retained 20,678 mouse genes, 19,914 GSE87466 human genes and 21,406 GSE75214 human genes. Of 17,091 strictly mapped one-to-one pairs, 12,109 were measured in all three datasets and formed the fixed scoring universe. All 42 experimental samples and all 193 human samples in the two primary comparisons were retained; no sample was removed on the basis of expression quality or separation by group. Every planned directional set contained the requested number of genes.

The experiment-2 anti-IL-17A signature scored lower in UC than controls in both cohorts: -0.022 (-0.034 to -0.009) in GSE87466 and -0.015 (-0.030 to -0.001) in GSE75214 (BH-adjusted P = 0.0010 and 0.0454). Experiment-2 anti-IL-17RA was also lower in both. Experiment-1 anti-IL-17RA was not clearly different in GSE87466 (0.005 (-0.022 to 0.033)), but was higher in GSE75214 (0.039 (0.027 to 0.050)). Thus, the IL-17 comparisons did not support a consistent UC-like transcriptional signature across experiments and cohorts (**Table 5**; **Figure 5**).

**Table 5:** Experimental-signature scores in external human UC cohorts.

Mouse intervention	Human cohort	UC − control (95% CI)	BH P
Anti-IL-17A (E2)	GSE87466	-0.022 (-0.034 to -0.009)	0.0010
Anti-IL-17A (E2)	GSE75214	-0.015 (-0.030 to -0.001)	0.0454
Anti-IL-17RA (E2)	GSE87466	-0.056 (-0.067 to -0.046)	8.22e-18
Anti-IL-17RA (E2)	GSE75214	-0.078 (-0.098 to -0.058)	5.05e-11
Anti-IL-17RA (E1)	GSE87466	0.005 (-0.022 to 0.033)	0.6943
Anti-IL-17RA (E1)	GSE75214	0.039 (0.027 to 0.050)	5.97e-09
Anti-IL-23p19 (E1)	GSE87466	-0.091 (-0.110 to -0.073)	2.55e-16
Anti-IL-23p19 (E1)	GSE75214	-0.116 (-0.129 to -0.102)	5.33e-28
Anti-IL-12/23p40 (E1)	GSE87466	-0.077 (-0.094 to -0.059)	1.02e-13
Anti-IL-12/23p40 (E1)	GSE75214	-0.091 (-0.103 to -0.079)	2.69e-24

Each signature comprises 250 mouse-up and 250 mouse-down genes selected within 12,109 common orthologs. Scores use within-sample percentile ranks; negative differences indicate lower alignment with the defined experimental direction in UC. GSE87466: 87 UC/21 controls; GSE75214: 74 active UC/11 controls. HC3 robust 95% t intervals use n − 2 degrees of freedom. BH adjustment covers all ten tests in this table. E1 and E2 denote mouse experiments 1 and 2. No clinical treatment effect is estimated.

**Figure 5:**
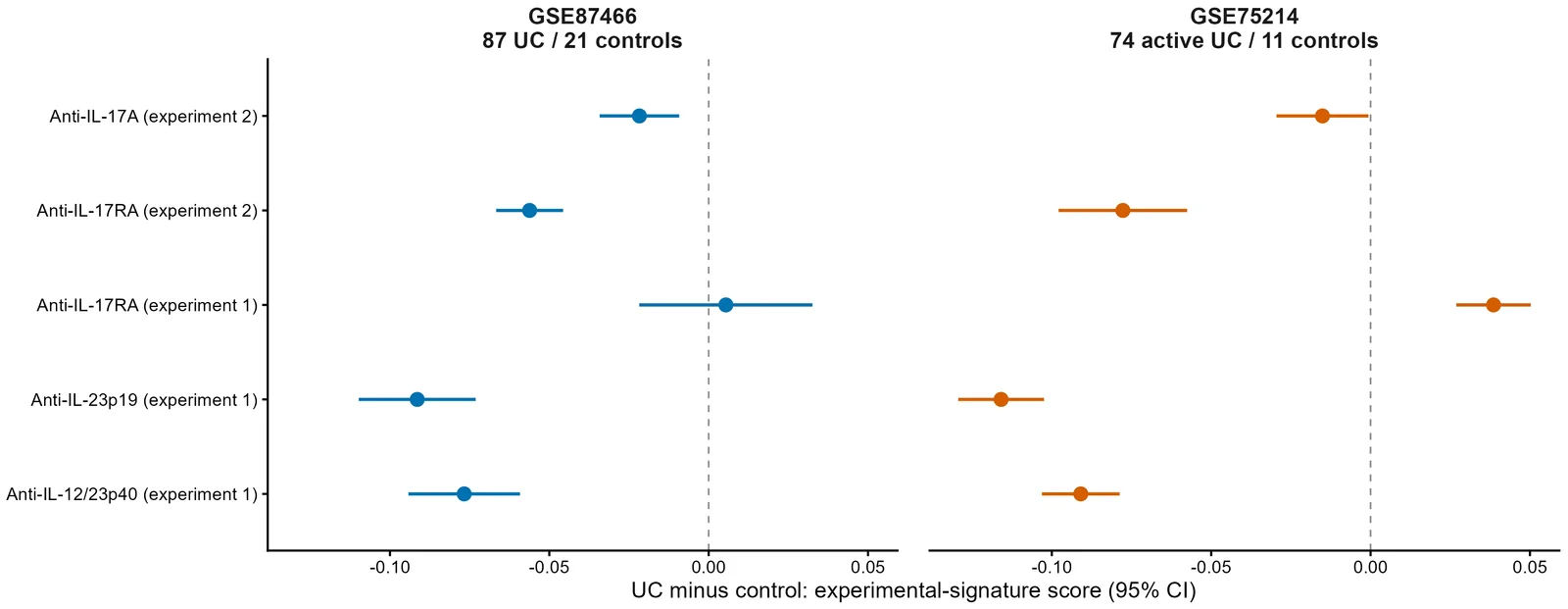
Experimental-signature comparison in human UC mucosa. Points show within-cohort UC-minus-control differences in the directional experimental-signature score; horizontal lines show HC3 robust 95% t intervals. Each mouse comparison is antibody versus the isotype group from the indicated experiment. Negative values indicate lower alignment with that mouse signature in UC than in controls. These are cross-sectional molecular comparisons of published data, not clinical effects of candidate drug exposure. All ten primary tests, including the inconsistent experiment-1 IL-17RA results, are shown. Full estimates and joint BH-adjusted P values appear in **Table 5**.

The anti-IL-23p19 and anti-IL-12/23p40 signatures scored lower in UC in both cohorts, with all four BH-adjusted P values below 1.1 × 10^-13^. The same pattern of lower scores for experiment-2 IL-17 blockade and both IL-23-pathway comparisons, and an uncertain GSE87466 experiment-1 IL-17RA result, was retained at both planned alternative signature sizes (**Supplementary Table S8**). Individual biopsy scores for all five primary experimental signatures in UC and control samples from both human cohorts are shown in **Supplementary Figure S2**. These score differences quantify alignment with defined gene sets; they are not drug-effect estimates or whole-transcriptome correlations. The full marker panel and all ten pharmacological profiles are available in **Supplementary Tables S9** and **S10**.

## Discussion

This event-centered study distinguishes persistent medication reporting associations from those strongly affected by recorded indication context. Seven candidates retained reporting support after excluding reports with a UC indication, whereas valganciclovir became imprecise. The external transcriptomic analysis examined whether experimental blockade of an implicated pathway produces a molecular pattern consistently represented in human UC mucosa. This pattern varied across IL-17 experiments, even though the FAERS association for secukinumab persisted. Combining these observations helps separate reporting evidence, clinical context, and molecular translation when choosing subsequent studies.

The observations involving secukinumab and bimekizumab have existing immunological and clinical context. In a randomized trial of 59 patients with active Crohn’s disease, secukinumab did not demonstrate efficacy and adverse events were more frequent than with placebo. That trial concerned established Crohn’s disease and does not directly establish a cause of new-onset UC. Bimekizumab inhibits IL-17A and IL-17F, and its FDA prescribing information includes a warning concerning new or worsening IBD. These findings support further investigation of intestinal effects of IL-17 inhibition but do not show that an individual FAERS report represents a drug-induced event or establish comparative risk between IL-17 inhibitors (12, 13).

The experimental study underlying GSE72212 reported distinct intestinal effects of IL-17 and IL-23 pathway blockade. Our secondary comparison extends that question to expression ranking in two human UC cohorts. It does not reproduce a simple model in which every IL-17-blockade signature resembles UC: the experiment-2 signatures were lower in UC, whereas the experiment-1 IL-17RA signature differed between cohorts. The IL-23-pathway signatures were consistently lower. These observations indicate that the particular experimental and human tissue contexts matter when translating a pathway hypothesis. They do not overturn the reported animal phenotypes, establish protective clinical effects of IL-17 drugs, or resolve which cell populations account for intestinal injury (4).

Recorded-indication exclusion identifies associations such as valganciclovir that are sensitive to pre-existing disease context; directional experimental-to-human comparisons test whether an implicated drug class shares a uniform disease expression pattern. The divergent IL-17 findings narrow the mechanistic claim that can be made from the reporting signal and define a need for drug-exposed intestinal samples with documented timing and cell composition. Pharmacological heterogeneity limits extrapolation of the IL-17 findings to other candidate medications.

Epidemiological evidence concerning isotretinoin and IBD is mixed. A propensity score matched cohort study found no statistically significant association between isotretinoin and incident IBD, although acne itself was associated with IBD. Another matched study found no statistically significant overall increase in UC, but a transient association during the first six months, with a hazard ratio of 1.93 (95% CI 1.29–2.88). A Korean case-control study reported an adjusted association with IBD. Differences in populations, comparators, ascertainment, and follow-up may contribute to these results. The present reporting association cannot resolve these differences or establish causality (14–16).

Pentosan polysulfate sodium has relevant clinical observational evidence. Jung and colleagues described colonic symptoms and histological abnormalities in a small, selected cohort of long-term users, alongside a separate cross-sectional association with IBD diagnosis. Some presentations did not fit classic IBD, emphasizing the distinction between medication-associated colopathy and UC. In a case series of 30 patients with IBD who had used pentosan polysulfate, ten had colonic dysplasia and six underwent colectomy for endoscopically unresectable dysplasia. Dysplasia in that selected series is not a cancer incidence estimate. These studies support further investigation while leaving the causal relationship and its magnitude uncertain (17, 18).

Clinical context is particularly important for valganciclovir and encorafenib. Valganciclovir is used in managing cytomegalovirus infection, which can complicate severe or refractory IBD. Its co-reporting with UC may therefore reflect treatment of a complication of pre-existing disease. Our report-level indication findings and sensitivity result are compatible with that alternative explanation, although they cannot adjudicate it. Excluding recorded UC indications does not exclude unrecorded UC or cytomegalovirus-related confounding. A published series also described severe inflammatory colitis after combined encorafenib and binimetinib treatment in patients previously exposed to immune checkpoint inhibitors. That combination and prior-treatment context cannot isolate the effect of encorafenib or equate all inflammatory colitis with UC (19, 20).

The less frequently reported candidates require caution. A small number of reports can produce a large ratio when the eligible exposed background is small, as illustrated by calcium carbonate and pentosan polysulfate. Conversely, sparse or non-estimable findings for hydrochlorothiazide and erdosteine cannot demonstrate safety. Selection of the candidates in the initial screen requires exploratory interpretation of the signal flags. The analysis supplies reporting evidence and contextual detail; it does not justify prescribing restrictions on the basis of a signal estimate alone.

Several limitations remain. FAERS is affected by underreporting, stimulated reporting, incomplete fields, residual duplicate reporting, and outcome misclassification. Restriction to health professionals and PS drugs does not remove these biases. The source snapshot and terminology-release identifiers were not available, complete upstream deduplication and unit conversion could not be independently reproduced, and the exported outcome table retained one category per report. Broad reporting windows and evolving treatment use can alter the comparator. Missing or nonspecific indications leave residual confounding, while co-medication descriptions are not multivariable adjustment. Similar profiles under separate case identifiers cannot be adjudicated without narratives (3).

Timing summaries are conditional on usable linked dates; the recorded event date was not adjudicated as UC onset. Normal-approximation IC bounds may be unreliable for sparse counts. The external analysis used published bulk-tissue data from a genetically susceptible, infection-triggered mouse model and human cross-sectional biopsies. Differences in species, cell composition, treatment history, sampling and experimental conditions may affect the signatures. The two human cohorts lacked sufficient covariates for comparable age, sex and medication adjustment, and they did not identify patients exposed to the candidate IL-17 inhibitors. The signatures therefore test cross-species expression alignment, not the clinical adverse effect of a specific medicine.

## Conclusion

The integrated analysis identified eight supported UC reporting candidates, seven of which remained supported after recorded-indication exclusion. In external patient and experimental data, IL-17 blockade signatures did not consistently reproduce a UC-like colonic expression pattern. These findings support clinically contextualized follow-up of the reporting associations and specify the limits of a generalized transcriptional explanation for IL-17-related intestinal effects.

## Data Availability

The original contributions presented in the study are included in the article/**Supplementary Material**. Further inquiries can be directed to the corresponding authors.
