# Supplementary material for: Ulcerative colitis reporting across medications: FAERS analysis and cross-species transcriptomic triangulation
Source: Front Immunol. 2026 Oct 9;17:1827266. doi: 10.3389/fimmu.2026.1827266 (PMC13690716; doi:10.3389/fimmu.2026.1827266)
Supplement: Supplementary file 1 [file SupplementaryFile1.docx]

Supplementary methods and tables

This supplement describes the report-level FAERS analyses, data quality assessment, and exploratory comparisons of experimental transcriptional signatures with human ulcerative colitis (UC) mucosa. FAERS estimates refer to the provider-default extraction of 13 September 2026, restricted to reporting quarters 2004Q1–2024Q4 and health-professional reporters. Detailed methods and supporting results are presented in Supplementary Tables S1–S10 and Supplementary Figures S1 and S2.

# Supplementary Methods

## Contingency construction and uncertainty

For each generic candidate, a is the number of unique PS-exposed UC reports; E is the eligible exposed-report total; U is the UC-report total; and N is the full eligible background. We calculated b = E − a, c = U − a, and d = N − E − U + a. Exposure matching used identical exact-name vectors in cases and the background. UC was the reaction term COLITIS ULCERATIVE, not an indication term. Whole-report indication sensitivity subtracts the exact UC-indication subset from every cell.

ROR and PRR log-scale intervals follow main-text Table 2. Pearson chi-square equals N(ad−bc)²/[(a+b)(a+c)(b+d)(c+d)]. Fisher intervals are conditional exact 95% intervals from R fisher.test; their estimates may differ slightly from the unconditional cross-product ratio. No 0.5 correction was used. An empty exposure margin makes the association non-estimable.

## Bayesian information-component calculation

The Bate beta-binomial construction was implemented with Gould digamma/trigamma moment calculations, matching the public PhViD 1.0.8 BCPNN implementation with MC = FALSE. Define p1 = 1+a+b, p2 = 1+c+d, q1 = 1+a+c, q2 = 1+b+d, r1 = 1+a, and r2 = N−a−1+(N+2)²/(p1q1). If ψ and ψ1 denote the digamma and trigamma functions, respectively, the posterior IC mean and variance are:

Mean(IC) = [ψ(r1) − ψ(r1+r2) − ψ(p1) + ψ(p1+p2) − ψ(q1) + ψ(q1+q2)] / ln(2).

Var(IC) = [ψ1(r1) − ψ1(r1+r2) + ψ1(p1) − ψ1(p1+p2) + ψ1(q1) − ψ1(q1+q2)] / [ln(2)]².

IC025 = Mean(IC) − 1.959964 × sqrt[Var(IC)]. This is a specified normal-approximation bound, not an exact posterior quantile or an MGPS estimate. The IC posterior mean is not the unshrunk log2(observed/expected) value. The prior construction is part of this stated implementation rather than an empirically fitted gamma-Poisson mixture. Sparse-count approximation limits are retained. The calculated values were independently compared with the public PhViD implementation. Methodological references are Bate et al. (1998), DOI 10.1007/s002280050466; Gould (2003), DOI 10.1002/pds.771; and Evans et al. (2001), DOI 10.1002/pds.677.

## Data quality and missing information

The demographic export has one row per report; all related-table primaryid values link to that export. Completeness and uniqueness do not constitute an independent audit of the complete provider case-version history. The precise snapshot and MedDRA release were not returned. The fresh export was also compared with the saved 8 September export; all seven tables were content-identical after sorting and excluding the CSV export index. The date of the current extraction remains 13 September 2026.

Missing markers included blanks, NA/NULL, UNKNOWN/UNK, NOT REPORTED, and the provider’s literal missing sentinel. Numeric AGE and WT were not multiplied again by their retained unit-code factors. Year/kg interpretation is supported by the provider’s code and numeric patterns, but the upstream raw-to-normalized conversion cannot be fully verified. A separate restriction to retained YR and KG codes is supplied below. The outcome table stores one retained category per report; unrecorded additional outcomes cannot be recovered.

Therapy records were linked by primaryid and DRUG_SEQ. Complete valid dates were required, without partial-date imputation. The primary interval was report EVENT_DT minus medication START_DT; zero-day intervals were retained. Missing/incomplete/invalid dates, no matched therapy record, dates after FDA receipt, negative intervals, and multiple distinct starts made a report–generic pair unevaluable. All linked therapy rows had to satisfy the date rules. END_DT was not required, but end-before-start and dates before 1900 were checked as additional plausibility flags; none affected these target records. No usable interval exceeded ten years. Primary exclusion-status counts are presented in Supplementary Table S4.

# Supplementary Table S1 Report population and missingness

| **Counting unit** | **n** |
| --- | --- |
| Demographic rows | 11,352 |
| Unique report identifiers | 11,352 |
| Unique case identifiers | 11,352 |
| Case identifiers with multiple report identifiers | 0 |
| Drug rows | 80,889 |
| Report–drug-sequence pairs | 80,369 |
| UC reports without a PS drug | 1 |
| Reaction rows | 66,001 |
| Report–reaction-PT pairs | 65,970 |
| Indication rows | 46,127 |
| Therapy rows | 29,858 |
| Retained outcome rows | 11,352 |
| Reporting quarters represented | 84 |

| **Variable** | **Nonmissing n** | **Missing n** | **Missing %** |
| --- | --- | --- | --- |
| AGE | 7892 | 3460 | 30.48 |
| WT | 3466 | 7886 | 69.47 |
| SEX | 10155 | 1197 | 10.54 |
| EVENT_DT | 7384 | 3968 | 34.95 |
| FDA_DT | 11352 | 0 | 0.00 |
| REPORTER_COUNTRY | 11224 | 128 | 1.13 |
| OCCP_COD | 11352 | 0 | 0.00 |

Missingness percentages use N = 11,352. The EVENT_DT nonmissing count includes 1,726 partial dates; only 5,658 reports have a complete valid event date. Thus nonmissing date is not synonymous with a usable timing observation. Reporter country has 128 empty/missing values and a further 385 explicitly unspecified values, giving 513 missing-or-unspecified locations after harmonization.

| **Variable and interpretation** | **n** | **Median (IQR)** |
| --- | --- | --- |
| Age in years; provider-normalized export interpretation | 7892 | 43.0 (26.0–60.0) |
| Age in years; retained YR code only | 7663 | 43.0 (26.0–59.0) |
| Weight in kg; provider-normalized export interpretation | 3466 | 67.0 (54.0–81.0) |
| Weight in kg; retained KG code only | 3422 | 66.6 (53.6–81.0) |

# Supplementary Table S2 Frozen exact medication vocabulary

| **Generic medication** | **Exact query terms** |
| --- | --- |
| Bimekizumab | BIMEKIZUMAB; BIMEKIZUMAB BKZX; BIMZELX |
| Calcium carbonate | CALCICHEW; CALCIUM CARBONATE; CALTRATE; CARBOCAL; OSCAL; TUMS; TUMS ULTRA |
| Efalizumab | EFALIZUMAB; RAPTIVA |
| Encorafenib | BRAFTOVI; ENCORAFENIB |
| Erdosteine | ERDOMED; ERDOSTEINE; ERDOTIN |
| Hydrochlorothiazide | HCT; HCTZ; HIDROCLOROTIAZIDA; HYDROCHLOROTHIAZIDE; HYDROCHLORTHIAZIDE; HYDROCHOROTHIAZIDE; HYDRODIURIL |
| Isotretinoin | ABSORICA; ABSORICA LD; ACCUTANE; AMNESTEEM; CLARAVIS; ISOTRETINOIN; ZENATANE |
| Pentosan polysulfate | ELMIRON; PENTOSAN POLYSULFATE; PENTOSAN POLYSULFATE SODIUM; SODIUM PENTOSAN POLYSULFATE |
| Secukinumab | AIN457; COSENTYX; SECUKINUMAB; VERXANT |
| Valganciclovir | ROVALCYTE; VALCYTE; VALGANCICLOVIR; VALGANCICLOVIR HCL; VALGANCICLOVIR HYDROCHLORIDE; VALGANCICLOVIR TABLET; VALIXA |

The same vectors were applied to DRUGNAME OR prod_ai with ROLE_COD = PS in the target and background populations. No fuzzy matching was applied in the final count extraction. This query vocabulary additionally contains the regulator-verified ERDOTIN and ERDOMED aliases, which did not add a target PS report. A name match identifies recorded exposure, not attribution to a single active ingredient or elimination of combination-treatment effects.

RAPTIVA–efalizumab was verified with the FDA label at https://www.fda.gov/media/75713/download. ERDOTIN–erdosteine was verified in the official company SmPC at https://www.medicines.org.uk/emc/product/287/smpc, and ERDOMED–erdosteine in the NPRA technical evaluation at https://quest3plus.bpfk.gov.my/front-end/attachment/3051/pharma/515313/1596681903.pdf. The mapping does not claim complete coverage of every international product name.

# Supplementary Table S3 Full report-level cells and sensitivity estimates

## Main analysis

N = 7,300,798; UC reports = 11,352. Cells a/b/c/d are defined in Table 1.

| **Medication** | **a** | **b** | **c** | **d** |
| --- | --- | --- | --- | --- |
| Bimekizumab | 5 | 424 | 11,347 | 7,289,022 |
| Calcium carbonate | 7 | 243 | 11,345 | 7,289,203 |
| Efalizumab | 9 | 1,587 | 11,343 | 7,287,859 |
| Encorafenib | 12 | 1,891 | 11,340 | 7,287,555 |
| Erdosteine | 0 | 0 | 11,352 | 7,289,446 |
| Hydrochlorothiazide | 1 | 4,327 | 11,351 | 7,285,119 |
| Isotretinoin | 130 | 22,223 | 11,222 | 7,267,223 |
| Pentosan polysulfate | 11 | 404 | 11,341 | 7,289,042 |
| Secukinumab | 204 | 29,610 | 11,148 | 7,259,836 |
| Valganciclovir | 9 | 2,161 | 11,343 | 7,287,285 |

## Whole-report UC-indication exclusion

N = 7,261,409; UC reports = 4,639. Cells a/b/c/d are defined in Table 1.

| **Medication** | **a** | **b** | **c** | **d** |
| --- | --- | --- | --- | --- |
| Bimekizumab | 4 | 424 | 4,635 | 7,256,346 |
| Calcium carbonate | 6 | 234 | 4,633 | 7,256,536 |
| Efalizumab | 9 | 1,587 | 4,630 | 7,255,183 |
| Encorafenib | 12 | 1,891 | 4,627 | 7,254,879 |
| Erdosteine | 0 | 0 | 4,639 | 7,256,770 |
| Hydrochlorothiazide | 1 | 4,327 | 4,638 | 7,252,443 |
| Isotretinoin | 130 | 22,223 | 4,509 | 7,234,547 |
| Pentosan polysulfate | 11 | 403 | 4,628 | 7,256,367 |
| Secukinumab | 201 | 29,592 | 4,438 | 7,227,178 |
| Valganciclovir | 3 | 2,149 | 4,636 | 7,254,621 |

The excluded subset contained 39,389 background reports, including 6,713 UC reports. Subtraction was verified independently for each medication and each cell.

## Estimates after whole-report UC-indication exclusion

| **Medication** | **a** | **ROR (95% CI)** | **PRR (95% CI)** | **IC mean (IC025)** |
| --- | --- | --- | --- | --- |
| Bimekizumab | 4 | 14.77 (5.52–39.55) | 14.64 (5.52–38.85) | 1.83 (0.49) |
| Calcium carbonate | 6 | 40.16 (17.85–90.36) | 39.18 (17.77–86.39) | 2.50 (1.37) |
| Efalizumab | 9 | 8.89 (4.61–17.12) | 8.84 (4.61–16.97) | 2.23 (1.31) |
| Encorafenib | 12 | 9.95 (5.64–17.56) | 9.89 (5.62–17.40) | 2.50 (1.69) |
| Erdosteine | 0 | NE | NE | NE |
| Hydrochlorothiazide | 1 | 0.36 (0.05–2.57) | 0.36 (0.05–2.57) | -1.30 (-3.57) |
| Isotretinoin | 130 | 9.39 (7.88–11.18) | 9.34 (7.85–11.11) | 3.09 (2.84) |
| Pentosan polysulfate | 11 | 42.80 (23.50–77.96) | 41.69 (23.25–74.73) | 3.19 (2.34) |
| Secukinumab | 201 | 11.06 (9.60–12.75) | 10.99 (9.55–12.66) | 3.33 (3.13) |
| Valganciclovir | 3 | 2.18 (0.70–6.78) | 2.18 (0.70–6.77) | 0.56 (-0.94) |

Seven candidates retain all stated criteria. Valganciclovir no longer meets them; its Fisher conditional interval is 0.45–6.40. Hydrochlorothiazide remains below the minimum target count, and erdosteine remains non-estimable. Main-analysis estimates are in Table 4.

# Supplementary Table S4 Recorded time to onset for all candidates

| **Medication** | **PS reports** | **Usable n (%)** | **Median (IQR) days** |
| --- | --- | --- | --- |
| Bimekizumab | 5 | 3 (60.00%) | Not summarized |
| Calcium carbonate | 7 | 0 (0.00%) | Not summarized |
| Efalizumab | 9 | 3 (33.33%) | Not summarized |
| Encorafenib | 12 | 5 (41.67%) | 90 (58–259) |
| Erdosteine | 0 | 0 (NA) | Not summarized |
| Hydrochlorothiazide | 1 | 0 (0.00%) | Not summarized |
| Isotretinoin | 130 | 65 (50.00%) | 175 (31–593) |
| Pentosan polysulfate | 11 | 0 (0.00%) | Not summarized |
| Secukinumab | 204 | 72 (35.29%) | 92 (46–289.25) |
| Valganciclovir | 9 | 2 (22.22%) | Not summarized |

Usable percentages divide evaluable report–generic pairs by all qualifying PS pairs for that medication. Median/IQR summaries require at least five usable pairs by the stated reporting convention. NA indicates a zero denominator. Primary exclusion-status counts are shown below. The denominator is never the number of therapy rows. Encorafenib is a particularly sparse timing summary.

## Primary timing status across all ten candidates

| **Mutually exclusive primary status** | **Report–generic pairs** |
| --- | --- |
| No medication-linked therapy row | 135 |
| Missing event date | 34 |
| Partial event date | 40 |
| Missing treatment start date | 1 |
| Partial treatment start date | 13 |
| Event date before treatment start | 15 |
| Usable complete-date interval | 150 |
| Total report–generic pairs | 388 |

The primary-status ledger assigns one status per report–generic pair: 150 pairs were evaluable and 238 were excluded. A report can contribute to more than one medication, so these are not 388 distinct people. No additional primary exclusion category occurred in these records.

## Supplementary Figure S1 Recorded time to onset

Points show medians and horizontal lines interquartile ranges, not confidence intervals. Labels show usable/all PS-report denominators and percentages. Only medications with at least five usable pairs are plotted. Recorded timing does not independently establish causation or a clinical latency distribution.

# Supplementary Table S5 Recorded indication and co-medication context

| **Medication** | **PS reports** | **Any medication UC indication** | **Target-linked UC indication** | **Unknown target-linked indication** |
| --- | --- | --- | --- | --- |
| Bimekizumab | 5 | 1 | 0 | 1 |
| Calcium carbonate | 7 | 1 | 0 | 7 |
| Efalizumab | 9 | 0 | 0 | 0 |
| Encorafenib | 12 | 0 | 0 | 3 |
| Erdosteine | 0 | 0 | 0 | 0 |
| Hydrochlorothiazide | 1 | 0 | 0 | 1 |
| Isotretinoin | 130 | 0 | 0 | 43 |
| Pentosan polysulfate | 11 | 0 | 0 | 7 |
| Secukinumab | 204 | 3 | 0 | 42 |
| Valganciclovir | 9 | 6 | 0 | 4 |

Any-medication UC indication means the exact UC term anywhere in the report. Target-linked indications use primaryid plus DRUG_SEQ. Unknown target-linked indication includes absent, empty, and the stated unknown-indication terms. The UC-indication exclusion removes all report cells if UC appears anywhere, even if it describes another medication. Missing or unknown indications are otherwise retained.

| **UC report indication group** | **Reports** |
| --- | --- |
| UC and another non-unknown indication | 1,563 |
| UC without another non-unknown indication | 5,150 |
| Other non-unknown indication without UC | 3,645 |
| No non-unknown indication | 994 |

## Valganciclovir-linked recorded indications

| **Recorded indication** | **Reports** |
| --- | --- |
| Cytomegalovirus gastroenteritis | 1 |
| Cytomegalovirus infection | 1 |
| Cytomegalovirus test positive | 1 |
| Antiviral prophylaxis | 1 |
| Prophylaxis | 1 |
| Product used for unknown indication | 4 |

Six of nine reports contain UC as an indication for another medication; none links UC to valganciclovir. Only one report has no non-unknown indication anywhere, whereas four lack a non-unknown indication specifically for valganciclovir. These completeness measures answer different questions. The co-medication profiles include mesalamine, infliximab and other IBD therapies, corticosteroids, tacrolimus, and mycophenolate-related agents. These co-medication descriptions reflect co-reporting and do not constitute multivariable adjustment. Similar profiles under different case identifiers cannot establish or exclude duplicate patients.

# Supplementary Table S6 Reconciliation with the legacy output

| **Candidate** | **Legacy merged worksheet a** | **Revised unique PS UC reports** |
| --- | --- | --- |
| Bimekizumab | 4 | 5 |
| Calcium carbonate | 3 | 7 |
| Efalizumab | 9 | 9 |
| Encorafenib | 12 | 12 |
| Erdosteine | 7 | 0 |
| Hydrochlorothiazide | 25 | 1 |
| Isotretinoin | 100 | 130 |
| Pentosan polysulfate | 11 | 11 |
| Secukinumab | 611 | 204 |
| Valganciclovir | 7 | 9 |

Legacy values are transcribed from the original merged result worksheet, not endorsed as valid unique-report counts. The original manuscript also contained inconsistent narrative totals (for example, isotretinoin 110 in the text and 100 in the merged worksheet). The new counts come from a distinct documented retrieval with normalization and report-level counting; their differences cannot be assigned to any one correction or interpreted as change over calendar time.

| **Audit finding** | **Verified evidence** | **Revision action** |
| --- | --- | --- |
| Duplicate aggregation | 32 pairs of initial drug rows were identical except drug identifier; secukinumab 208 appeared twice and later became 416. | Replace aggregate summation with unique report–generic counts. |
| PRR intervals | All 353 legacy merged rows used the additive rather than exponentiated log-scale expression. | Recompute PRR and log-scale 95% intervals from verified cells. |
| IC and IC025 | Legacy IC was unshrunk log2(O/E); lower-bound/variance calculations were inconsistent with the specified posterior calculation. | Report posterior mean and approximate IC025 separately. |
| MGPS | Legacy EBGM matched O/E and no fitted empirical-Bayes mixture/posterior interval was demonstrated. | Withdraw MGPS/EBGM results and four-algorithm confirmation claim. |
| Former Figure 4 | A reproducible regression specification and supporting modeling population could not be established. | Withdraw the model and replace the figure with indication-sensitivity estimates. |

Table S6 summarizes the numerical and methodological discrepancies identified in the original analysis. Algorithm-only corrections on legacy cells are diagnostic and are not substituted for a valid report-level analysis. The external secondary transcriptomic analyses added to this revision are reported in Supplementary Tables S7–S10; they do not validate medication-specific clinical causality.

# Supplementary Table S7 External data and reproducible methods

| **Accession / platform** | **Primary analysis** | **Other deposited samples** |
| --- | --- | --- |
| GSE72212 / GPL11180 | 42 mouse colon samples; two experiments; five antibody–isotype contrasts | All groups retained within their own experiment |
| GSE87466 / GPL13158 | 87 adult UC + 21 healthy colon biopsies | 108 explicit unique subject identifiers; all included |
| GSE75214 / GPL6244 | 74 active UC colon + 11 normal colon biopsies | 23 inactive UC for descriptive context; 8 CD colon + 78 ileal samples excluded from primary comparison |

The source studies and GEO records were accessed on 13 September 2026. GSE87466 comprises screening-period adult biopsies associated with the PURSUIT program; healthy controls were separately recruited. GSE75214 distinguishes activity and biopsy site but does not supply sufficient subject-level demographics or treatment covariates for harmonized adjustment. GSE59071 overlaps its colon samples and is not independent replication. [1, 2, 3]

## Mouse groups and within-experiment comparisons

| **Contrast** | **Treated n** | **Isotype n** | **Total model n** |
| --- | --- | --- | --- |
| IL17RA_exp1 | 5 | 3 | 22 |
| p19_exp1 | 5 | 3 | 22 |
| p40_exp1 | 3 | 3 | 22 |
| IL17A_exp2 | 5 | 4 | 20 |
| IL17RA_exp2 | 5 | 4 | 20 |

The model uses Abcb1a-deficient mice challenged with H. bilis on days 0 and 7; antibody treatment began on day 0 according to the deposited metadata. Experiment 1 includes naive, PBS, isotype, anti-p19, anti-p40 and anti-IL-17RA groups; experiment 2 includes naive, PBS, isotype, anti-IL-17A and anti-IL-17RA. Exact antibody clone, dose, later dosing schedule, terminal sampling day, sex and age were not recovered from the accessible deposition and are not inferred. No new animal experiment was undertaken.

## Preprocessing and orthology

GSE72212 has 45,141 positive MAS5 probe intensities (range 3.47–16,887.64). After log2 transformation, quantile normalization was performed within each experiment. Human matrices were deposited RMA log2 values and were not transformed or normalized again. GSM-to-column identity was checked explicitly. Multi-gene probes, empty assignments, controls and nonstandard family/clone labels were excluded. Among probes with a unique gene, the highest all-deposited-sample mean was retained, with probe identifier breaking exact ties. The all-sample rule includes non-primary samples in GSE75214 and uses no disease effect or P value. Expression distributions, principal components and sample membership were examined; no sample was excluded after quality control.

The MGI report was downloaded from its official server (source last-modified 7 September 2026). A class had to contain exactly one human and one mouse gene; each symbol and gene identifier also had to occur in only that class across the complete raw report. This excluded mappings ambiguous in another class, even when that other class was itself ineligible. The resulting 17,091 pairs yielded 12,109 measured genes common to all datasets. Gene symbols were joined using the downloaded mappings with preserved case, not converted between species by capitalization. [4]

## Supplementary methods: directional scoring

Separate no-intercept group models in limma 3.62.2 used all groups from the respective mouse experiment. Empirical-Bayes estimation used trend = FALSE and robust = FALSE. Each of the five contrasts is antibody-treated minus mIgG, and all-gene log2 fold changes, moderated t statistics, P values and within-contrast BH values were calculated. Separate human models compare UC with Control. No missing age, sex or medication value was imputed, and no adjustment for these unavailable covariates was claimed. [5]

Within the fixed common ortholog universe, positive moderated t values were sorted from highest to lowest and negative values from lowest to highest; exact ties were resolved by human gene symbol. The first 250 in each direction formed the primary set. All sets met the requested size. For G = 12,109 measured genes in a human sample, percentile rank = (average rank − 1)/(G − 1). The signature score is the mean percentile rank of mouse-up genes minus that of mouse-down genes. It lies between −1 and 1. Selection depends only on the mouse contrast; human UC labels and results do not select signature genes.

Each human cohort used score = intercept + coefficient × UC indicator. HC3 covariance is (XᵀX)⁻¹ Xᵀ diag[eᵢ²/(1−hᵢ)²] X (XᵀX)⁻¹, where eᵢ is the residual and hᵢ the leverage. Two-sided P values and 95% intervals use a t distribution with n−2 degrees of freedom. The coefficient equals mean(UC) minus mean(Control). An independent two-group variance identity was checked for every result. Benjamini–Hochberg adjustment covers the ten primary tests jointly. The 100- and 500-gene-per-direction analyses comprise a separate family of twenty tests; none replaced the primary size.

This was an exploratory analysis added during revision. The source abstracts and experimental rationale were known before analysis. The analysis plan was fixed before differential expression and patient scores were calculated. It is not a prospective registration. All planned comparisons and alternative sizes are reported. Inactive UC samples were retained only for descriptive context, without additional hypothesis tests. Comparisons between different signature coefficients were not formally tested.

# Supplementary Table S8 All planned external score comparisons

## Primary: 250 genes per direction

| **Contrast** | **Human cohort** | **Difference (95% CI)** | **Raw P** | **BH P** |
| --- | --- | --- | --- | --- |
| IL17A_exp2 | GSE87466 | -0.022 (-0.034 to -0.009) | 8.05e-04 | 0.0010 |
| IL17A_exp2 | GSE75214 | -0.015 (-0.030 to -0.001) | 0.0408 | 0.0454 |
| IL17RA_exp2 | GSE87466 | -0.056 (-0.067 to -0.046) | 2.47e-18 | 8.22e-18 |
| IL17RA_exp2 | GSE75214 | -0.078 (-0.098 to -0.058) | 3.03e-11 | 5.05e-11 |
| IL17RA_exp1 | GSE87466 | 0.005 (-0.022 to 0.033) | 0.6943 | 0.6943 |
| IL17RA_exp1 | GSE75214 | 0.039 (0.027 to 0.050) | 4.18e-09 | 5.97e-09 |
| p19_exp1 | GSE87466 | -0.091 (-0.110 to -0.073) | 1.02e-16 | 2.55e-16 |
| p19_exp1 | GSE75214 | -0.116 (-0.129 to -0.102) | 5.33e-29 | 5.33e-28 |
| p40_exp1 | GSE87466 | -0.077 (-0.094 to -0.059) | 5.08e-14 | 1.02e-13 |
| p40_exp1 | GSE75214 | -0.091 (-0.103 to -0.079) | 5.38e-25 | 2.69e-24 |

Control/UC counts are 21/87 for GSE87466 and 11/74 for GSE75214. Every set contains the stated size in each direction. Negative differences indicate lower scores in UC. The primary family has 10 tests; both sensitivity sizes share one 20-test BH family.

## Sensitivity: 100 genes per direction

| **Contrast** | **Human cohort** | **Difference (95% CI)** | **Raw P** | **BH P** |
| --- | --- | --- | --- | --- |
| IL17A_exp2 | GSE87466 | -0.035 (-0.049 to -0.021) | 3.17e-06 | 4.22e-06 |
| IL17A_exp2 | GSE75214 | -0.025 (-0.040 to -0.011) | 0.0010 | 0.0012 |
| IL17RA_exp2 | GSE87466 | -0.069 (-0.082 to -0.056) | 8.68e-18 | 2.17e-17 |
| IL17RA_exp2 | GSE75214 | -0.088 (-0.105 to -0.070) | 3.96e-16 | 8.80e-16 |
| IL17RA_exp1 | GSE87466 | -0.004 (-0.033 to 0.025) | 0.7996 | 0.7996 |
| IL17RA_exp1 | GSE75214 | 0.035 (0.023 to 0.047) | 9.83e-08 | 1.40e-07 |
| p19_exp1 | GSE87466 | -0.125 (-0.144 to -0.105) | 5.61e-23 | 2.81e-22 |
| p19_exp1 | GSE75214 | -0.166 (-0.184 to -0.148) | 3.59e-31 | 7.18e-30 |
| p40_exp1 | GSE87466 | -0.104 (-0.123 to -0.084) | 1.68e-18 | 5.59e-18 |
| p40_exp1 | GSE75214 | -0.143 (-0.159 to -0.126) | 1.27e-28 | 1.27e-27 |

Control/UC counts are 21/87 for GSE87466 and 11/74 for GSE75214. Every set contains the stated size in each direction. Negative differences indicate lower scores in UC. The primary family has 10 tests; both sensitivity sizes share one 20-test BH family.

## Sensitivity: 500 genes per direction

| **Contrast** | **Human cohort** | **Difference (95% CI)** | **Raw P** | **BH P** |
| --- | --- | --- | --- | --- |
| IL17A_exp2 | GSE87466 | -0.023 (-0.035 to -0.011) | 1.51e-04 | 1.89e-04 |
| IL17A_exp2 | GSE75214 | -0.018 (-0.030 to -0.006) | 0.0038 | 0.0042 |
| IL17RA_exp2 | GSE87466 | -0.056 (-0.066 to -0.046) | 1.97e-18 | 5.62e-18 |
| IL17RA_exp2 | GSE75214 | -0.068 (-0.087 to -0.050) | 1.96e-10 | 3.57e-10 |
| IL17RA_exp1 | GSE87466 | 0.004 (-0.019 to 0.027) | 0.7273 | 0.7656 |
| IL17RA_exp1 | GSE75214 | 0.030 (0.020 to 0.040) | 3.71e-08 | 5.70e-08 |
| p19_exp1 | GSE87466 | -0.070 (-0.087 to -0.054) | 2.22e-13 | 4.43e-13 |
| p19_exp1 | GSE75214 | -0.094 (-0.106 to -0.082) | 5.05e-27 | 3.36e-26 |
| p40_exp1 | GSE87466 | -0.058 (-0.077 to -0.039) | 1.42e-08 | 2.36e-08 |
| p40_exp1 | GSE75214 | -0.072 (-0.083 to -0.061) | 1.33e-22 | 5.31e-22 |

Control/UC counts are 21/87 for GSE87466 and 11/74 for GSE75214. Every set contains the stated size in each direction. Negative differences indicate lower scores in UC. The primary family has 10 tests; both sensitivity sizes share one 20-test BH family.

# Supplementary Figure S2 Individual external signature scores

Each point is one human biopsy; boxes show median and interquartile range, with whiskers extending to observations within 1.5 interquartile ranges. All individual points, including extremes, are shown. Scores use the primary 250-up/250-down signatures. The two human cohorts are analyzed separately. Negative or positive raw scores depend on the particular signature and do not represent disease probabilities.

# Supplementary Table S9 Fixed epithelial and inflammatory marker panel

## Human UC versus control contrasts

| **Human symbol** | **Dataset / contrast** | **Log2 FC** | **BH P** |
| --- | --- | --- | --- |
| PIGR | GSE87466 | -0.055 | 0.2320 |
| MUC2 | GSE87466 | 0.190 | 0.5493 |
| TJP1 | GSE87466 | -0.243 | 0.0372 |
| OCLN | GSE87466 | -0.624 | 5.65e-05 |
| CLDN1 | GSE87466 | 1.488 | 1.32e-11 |
| CXCL1 | GSE87466 | 3.815 | 1.71e-24 |
| CXCL2 | GSE87466 | 3.033 | 1.50e-17 |
| LCN2 | GSE87466 | 2.833 | 5.98e-28 |
| PIGR | GSE75214 | -0.366 | 0.3127 |
| MUC2 | GSE75214 | 0.193 | 0.6747 |
| TJP1 | GSE75214 | -0.471 | 1.69e-09 |
| OCLN | GSE75214 | Not mapped | NA |
| CLDN1 | GSE75214 | 2.230 | 6.79e-12 |
| CXCL1 | GSE75214 | 3.454 | 9.61e-23 |
| CXCL2 | GSE75214 | 2.375 | 9.17e-17 |
| LCN2 | GSE75214 | 3.527 | 1.60e-16 |

All eight predefined genes are retained. Positive log2 FC means higher UC expression in the human comparison or higher antibody-treated expression in the mouse comparison. BH values are from each full gene-level contrast, not corrected only within this selected marker panel. In the human panel, Not mapped indicates no uniquely eligible probe-to-gene assignment; GSE75214 OCLN probe 8105908 maps jointly to OCLN and LOC647859. Neither denotes absent expression. Marker values are contextual and were not used to select the signatures.

## Mouse intervention contrasts

| **Human symbol** | **Dataset / contrast** | **Log2 FC** | **BH P** |
| --- | --- | --- | --- |
| CLDN1 | IL17A_exp2 | -0.205 | 0.6412 |
| CXCL1 | IL17A_exp2 | Not mapped | NA |
| CXCL2 | IL17A_exp2 | Not mapped | NA |
| LCN2 | IL17A_exp2 | -0.581 | 0.7522 |
| MUC2 | IL17A_exp2 | -0.080 | 0.9357 |
| OCLN | IL17A_exp2 | 0.433 | 0.1338 |
| PIGR | IL17A_exp2 | -0.993 | 0.0604 |
| TJP1 | IL17A_exp2 | 0.451 | 0.0247 |
| CLDN1 | IL17RA_exp2 | -0.543 | 0.1574 |
| CXCL1 | IL17RA_exp2 | Not mapped | NA |
| CXCL2 | IL17RA_exp2 | Not mapped | NA |
| LCN2 | IL17RA_exp2 | -0.446 | 0.7987 |
| MUC2 | IL17RA_exp2 | -0.433 | 0.5100 |
| OCLN | IL17RA_exp2 | 0.253 | 0.4272 |
| PIGR | IL17RA_exp2 | -0.778 | 0.1731 |
| TJP1 | IL17RA_exp2 | 0.215 | 0.3076 |
| CLDN1 | IL17RA_exp1 | 0.649 | 0.2560 |
| CXCL1 | IL17RA_exp1 | Not mapped | NA |
| CXCL2 | IL17RA_exp1 | Not mapped | NA |
| LCN2 | IL17RA_exp1 | 0.293 | 0.7288 |
| MUC2 | IL17RA_exp1 | 0.002 | 0.9976 |
| OCLN | IL17RA_exp1 | -0.009 | 0.9829 |
| PIGR | IL17RA_exp1 | -0.494 | 0.1966 |
| TJP1 | IL17RA_exp1 | 0.398 | 0.1044 |
| CLDN1 | p19_exp1 | 1.159 | 0.0159 |
| CXCL1 | p19_exp1 | Not mapped | NA |
| CXCL2 | p19_exp1 | Not mapped | NA |
| LCN2 | p19_exp1 | -5.712 | 3.94e-08 |
| MUC2 | p19_exp1 | 0.686 | 0.0717 |
| OCLN | p19_exp1 | 0.592 | 0.0203 |
| PIGR | p19_exp1 | 1.072 | 0.0016 |
| TJP1 | p19_exp1 | 0.633 | 0.0029 |
| CLDN1 | p40_exp1 | 0.882 | 0.1278 |
| CXCL1 | p40_exp1 | Not mapped | NA |
| CXCL2 | p40_exp1 | Not mapped | NA |
| LCN2 | p40_exp1 | -5.410 | 1.48e-06 |
| MUC2 | p40_exp1 | 0.641 | 0.1707 |
| OCLN | p40_exp1 | 0.274 | 0.4087 |
| PIGR | p40_exp1 | 0.994 | 0.0114 |
| TJP1 | p40_exp1 | 0.436 | 0.0759 |

All eight predefined genes are retained. Positive log2 FC means higher UC expression in the human comparison or higher antibody-treated expression in the mouse comparison. BH values are from each full gene-level contrast, not corrected only within this selected marker panel. In the mouse panel, Not mapped indicates no eligible measured one-to-one ortholog. Neither denotes absent expression. Marker values are contextual and were not used to select the signatures.

# Supplementary Table S10 Pharmacological evidence for all ten candidates

| **Candidate** | **Strict direct human target(s)** | **Pharmacological classification** |
| --- | --- | --- |
| Bimekizumab | IL17A;IL17F | direct human primary |
| Calcium carbonate | Not assigned | chemical or physiological action |
| Efalizumab | ITGAL | direct human primary |
| Encorafenib | BRAF | direct human primary |
| Erdosteine | Not assigned | active metabolite nonspecific chemical action |
| Hydrochlorothiazide | SLC12A3 | direct human primary |
| Isotretinoin | Not assigned | prodrug and context dependent receptor mechanism |
| Pentosan polysulfate | Not assigned | incompletely understood nonspecific action |
| Secukinumab | IL17A | direct human primary |
| Valganciclovir | Not assigned | active metabolite viral target |

Five candidates have curated direct primary human targets, yielding six drug–gene edges across five unique genes. Isotretinoin is separated from retinoic-acid metabolite/receptor action; valganciclovir acts through an active antiviral metabolite and viral enzymes; mineral/antacid and other non-specific actions are not converted into arbitrary human targets. Missing direct-target assignments indicate the scope of this curation rather than proof of no biological interaction. Because direct human target coverage was limited, an all-drug protein-network enrichment was not performed. IL-17RA is receptor context for the IL-17-neutralizing clinical antibodies; the experimental receptor-blockade data do not establish that clinical secukinumab or bimekizumab was administered.

## Bimekizumab

Neutralizes human IL-17A, IL-17F, and IL-17A/F cytokines. The A/F heterodimer involves the same two genes. Two direct cytokine targets. Human clinical drug is not equivalent to an experimental anti-mouse IL-17RA antibody.

Source: https://www.accessdata.fda.gov/drugsatfda_docs/label/2024/761151s010lbl.pdf; https://www.ebi.ac.uk/chembl/explore/compound/CHEMBL4297700. Location: FDA section 12.1; ChEMBL mechanism IDs 7924 and 8132.

## Calcium carbonate

Calcium supplementation and antacid chemistry. Gastric acid converts carbonate salt to calcium chloride; absorbed calcium serves general mineral physiology. Empty ChEMBL mechanism lookup is a coverage fact, not proof that no biological interaction exists.

Source: https://www.medicines.org.uk/emc/product/1295/smpc; https://www.ebi.ac.uk/chembl/explore/compound/CHEMBL1200539. Location: SmPC sections 5.1 and 5.2; ChEMBL exact molecule search and empty mechanism result.

## Efalizumab

Binds CD11a, the alpha-L subunit of LFA-1. LFA-1 is the ITGAL/ITGB2 complex; inhibition alters leukocyte adhesion and activation. ChEMBL target is a protein complex, but its binding-site comment specifically identifies the alpha subunit.

Source: https://www.accessdata.fda.gov/drugsatfda_docs/label/2009/125075s130lbl.pdf; https://www.ebi.ac.uk/chembl/explore/compound/CHEMBL1201575. Location: FDA Clinical Pharmacology / Mechanism of Action; ChEMBL mechanism 2042 binding-site comment.

## Encorafenib

Kinase inhibition with BRAF V600E therapeutic context. FDA cell-free IC50: BRAF V600E 0.35 nM, wild-type BRAF 0.47 nM, CRAF 0.3 nM. FDA also reports in-vitro binding to JNK1/JNK2/JNK3/LIMK1/LIMK2/MEK4/STK36 at clinically achievable concentrations. ChEMBL comment says little/no wild-type BRAF activity; FDA gives explicit cell-free wild-type inhibition. These differences in assay conditions and context do not establish mutation-exclusive biochemical binding. Wild-type and mutant BRAF remain one gene.

Source: https://www.accessdata.fda.gov/drugsatfda_docs/label/2024/210496s016lbl.pdf; https://www.ebi.ac.uk/chembl/explore/compound/CHEMBL3301612. Location: FDA section 12.1; ChEMBL mechanism 5298, V600E variant.

## Erdosteine

Prodrug yielding free-thiol metabolites with mucolytic and antioxidant effects. Active M1 metabolite disrupts bronchial mucoprotein disulfide bonds; no single protein primary target specified. ChEMBL direct_interaction=1 with a null target is insufficient to assign a gene.

Source: https://www.medicines.org.uk/emc/product/287/smpc; https://www.ebi.ac.uk/chembl/explore/compound/CHEMBL1697744. Location: SmPC sections 5.1 and 5.2; ChEMBL mechanisms 8257 and 8563 have null target IDs.

## Hydrochlorothiazide

Inhibits the thiazide-sensitive sodium-chloride cotransporter NCC. Human NCC co-structure with hydrochlorothiazide and functional inhibition are reported in PMID 39143061.

Source: https://eutils.ncbi.nlm.nih.gov/entrez/eutils/efetch.fcgi?db=pubmed&id=10951254,16575387,39143061&retmode=xml; https://www.ebi.ac.uk/chembl/explore/compound/CHEMBL435. Location: PMID 39143061 abstract; ChEMBL mechanism 350 and target CHEMBL1876.

## Isotretinoin

Retinoid with incompletely established acne mechanism; intracellular isomerization can mediate RAR effects. ChEMBL assigns the RAR family and explicitly comments Prodrug. Human sebocyte experiments support intracellular conversion to all-trans retinoic acid (PMID10951254); a separate SEB-1 model found RAR-independent apoptosis (PMID16575387). These findings provide context for retinoid-receptor biology but do not establish a single receptor-mediated mechanism. The direct_interaction flag conflicts with the prodrug/family abstraction and cannot establish isoform-resolved parent-drug binding.

Source: https://www.accessdata.fda.gov/drugsatfda_docs/label/2019/021951s013lbl.pdf;https://eutils.ncbi.nlm.nih.gov/entrez/eutils/efetch.fcgi?db=pubmed&id=10951254,16575387,39143061&retmode=xml; https://www.ebi.ac.uk/chembl/explore/compound/CHEMBL547. Location: FDA section 12.1; ChEMBL mechanism 2365; PMID10951254 and PMID16575387.

## Pentosan polysulfate

Heparin-like polysaccharide; mechanism in interstitial cystitis remains unknown. Anticoagulant/fibrinolytic actions and proposed bladder-wall coating are mechanistic context. The curated molecule record refers specifically to pentosan polysulfate sodium.

Source: https://dailymed.nlm.nih.gov/dailymed/fda/fdaDrugXsl.cfm?setid=f0ba651e-3d8a-11df-8fbe-119855d89593&type=display; https://www.ebi.ac.uk/chembl/explore/compound/CHEMBL1201516. Location: DailyMed Clinical Pharmacology; ChEMBL mechanism 2120 has null target ID.

## Secukinumab

Neutralizes the human IL-17A cytokine. Prevents interaction with the IL-17 receptor complex. Shared target with bimekizumab creates nonindependent drug-target edges; it is one unique gene in a union.

Source: https://www.accessdata.fda.gov/drugsatfda_docs/label/2024/125504s080lbl.pdf; https://www.ebi.ac.uk/chembl/explore/compound/CHEMBL1743068. Location: FDA section 12.1; ChEMBL mechanism 2327.

## Valganciclovir

Prodrug of ganciclovir; ganciclovir triphosphate inhibits CMV DNA polymerase pUL54. CMV pUL97 catalyses initial phosphorylation; host esterases and kinases contribute activation/metabolism. The parent valganciclovir ChEMBL mechanism query is empty. The ganciclovir ChEMBL record concerns human herpesvirus 1 polymerase, rather than CMV polymerase or a human protein. CMV pUL54 attribution is based on the prescribing information.

Source: https://dailymed.nlm.nih.gov/dailymed/fda/fdaDrugXsl.cfm?setid=2e8a4d62-2098-4ffe-a154-e45cfdfe6c4c&type=display; https://www.ebi.ac.uk/chembl/explore/compound/CHEMBL1201314. Location: DailyMed sections 12.1, 12.3, and 12.4 Microbiology.

# Supplementary references

1. Maxwell JR, Zhang Y, Brown WA, Smith CL, Byrne FR, Fiorino M, et al. Differential Roles for Interleukin-23 and Interleukin-17 in Intestinal Immunoregulation. Immunity. 2015;43(4):739-50. doi: 10.1016/j.immuni.2015.08.019.
2. Li K, Strauss R, Ouahed J, Chan D, Telesco SE, Shouval DS, et al. Molecular Comparison of Adult and Pediatric Ulcerative Colitis Indicates Broad Similarity of Molecular Pathways in Disease Tissue. J Pediatr Gastroenterol Nutr. 2018;67(1):45-52. doi: 10.1097/MPG.0000000000001898.
3. Vancamelbeke M, Vanuytsel T, Farré R, Verstockt S, Ferrante M, Van Assche G, et al. Genetic and Transcriptomic Bases of Intestinal Epithelial Barrier Dysfunction in Inflammatory Bowel Disease. Inflamm Bowel Dis. 2017;23(10):1718-1729. doi: 10.1097/MIB.0000000000001246.
4. Mouse Genome Informatics. Mouse–human homology sequence report (HOM_MouseHumanSequence.rpt). 2026. Available from: https://www.informatics.jax.org/downloads/reports/HOM_MouseHumanSequence.rpt (accessed 2026 Sep 13).
5. Ritchie ME, Phipson B, Wu D, Hu Y, Law CW, Shi W, et al. limma powers differential expression analyses for RNA-sequencing and microarray studies. Nucleic Acids Res. 2015;43(7):e47. doi: 10.1093/nar/gkv007.
